# A Case of Cardiac Amyloidosis Initially Misdiagnosed as Syndrome X

**DOI:** 10.4021/cr67w

**Published:** 2011-07-25

**Authors:** Hyung Rae Sohn, Bong Gun Song, Seong Yeon Jeong, Su-Min Hong, Hyun Gul Jung, Hye-Jin Jung, Wook-Hyun Cho, Suk-Koo Choi

**Affiliations:** a Division of Cardiology, Cardiac and Vascular Center, Department of Medicine, Inje University Seoul Paik Hospital, Inje University School of Medicine, Seoul, Korea

**Keywords:** Cardiac amyloidosis, Syndrome X, Heart transplantation

## Abstract

Cardiac infiltration of amyloid fibril results in progressive cardiomyopathy with a grave prognosis and results in cardiac diseases such as congestive heart disease, cardiomyopathy, valvular heart disease, and arrhythmias. We present a rare case of cardiac amyloidosis initially misdiagnosed as syndrome X in which recurrent chest pain and progressive heart failure could be managed finally by heart transplantation.

## Introduction

Amyloidosis is a clinical disorder caused by extracellular deposition of insoluble abnormal fibrils, derived from aggregation of misfolded normally soluble protein [1-4]. Cardiac infiltration of amyloid fibril results in progressive cardiomyopathy with a grave prognosis and results in cardiac diseases such as congestive heart disease, cardiomyopathy, valvular heart disease, and arrhythmias [1-4]. A prominent clinical feature of cardiac amyloidosis is a syndrome of heart failure, characterized by restrictive hemodynamics and progressive deterioration of systolic function [1-4]. Here, we reported an interesting case of cardiac amyloidosis initially misdiagnosed as syndrome X and we discussed the key findings of the disease along with the latest evidence regarding the management and prognosis of cardiac amyloidosis.

## Case Report

**Figure 1:**
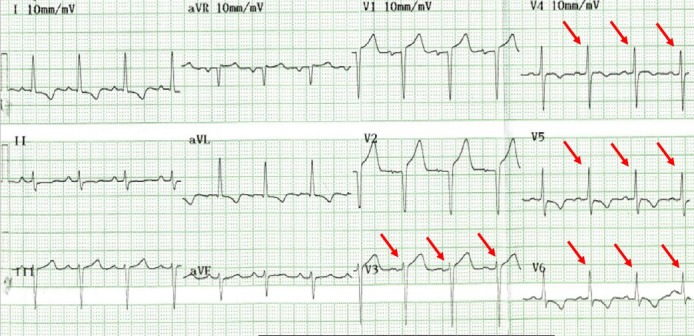
The electrocardiogram showed low voltage in the limb leads and a small R wave amplitude across the precordial leads (arrows).

**Figure 2:**
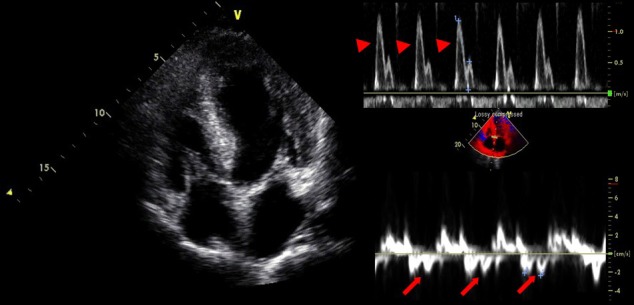
The septal and free walls of the left ventricle were mildly thickened, and the left ventricular cavity dimension was enlarged. Doppler analysis of transmitral inflow showed restrictive pattern with high E/A ratio (2.3) (arrowheads). Tissue Doppler of mitral annular diastolic velocity (E’) was markedly re­duced (2 cm/s) (arrows).

**Figure 3:**
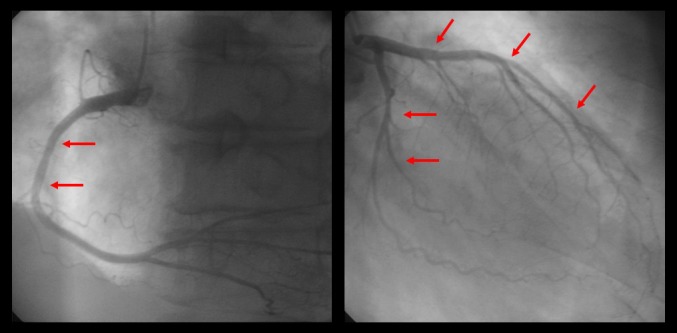
Repeated coronary angiography did not show any significant stenosis of the coronary arteries (arrows).

**Figure 4:**
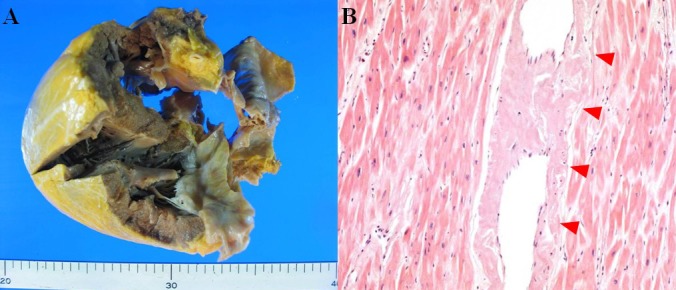
The gross finding showed that deposition of pale staining amorphous material in blood vessel and interstitium with degenerated myofibers (A) and microscopic findings stained with hematoxylin and eosin revealed myxoid and amorphous deposits in perivascular and interstitial spaces (B, arrowheads).

**Figure 5:**
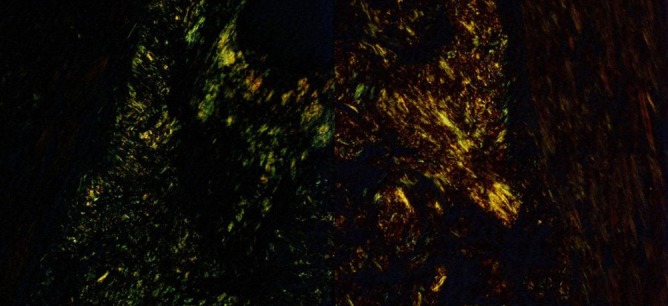
Congo-red stain of the specimen revealed apple green birefringence under polarized microscopy.

A 77-year-old man was admitted to our hospital with chest pain and dyspnea on exertion. He reported recurrent episodes of chest pain three years previously. Because the exercise treadmill test showed ST segment depression on the electrocardiogram, he was suspected of having coronary artery disease. Left ventricular function was well preserved with no wall motion abnormalities as assessed by echocardiography. Coronary angiography showed no significant stenosis of any of the epicardial coronary arteries. The provocation test with ergonovine was performed to rule out coronary vasospasm, and a negative result was obtained. Therefore, the patient was diagnosed as having cardiac syndrome X and has been managed medically. On admission, the electrocardiography showed low voltage in the limb leads and a small R wave amplitude across the precordial leads (Fig. 1, arrows). The chest roentgenogram demonstrated moderate cardiomegaly with interstitial congestion and marked pleural effusion in the both lung field. Initial laboratory studies revealed normal ranges of renal function test and liver function test, mildly elevated troponin-I (0.15 ng/mL: normal < 0.1 ng/mL), and markedly elevated BNP (5643 pg/mL: normal < 100 pg/mL). On echocardiography, the septal and free walls of the left ventricle were mildly thickened, and the left ventricu­lar cavity dimension was enlarged (Fig. 2). Left systolic function was severely decreased with global hypokinesia, with an estimated ejection fraction in the 29% range. Doppler analysis of transmitral inflow showed restrictive pattern with high E/A ratio (2.3) (Fig. 2, arrowheads). Tissue Doppler of mitral annular diastolic velocity (E’) was markedly reduced (2 cm/s) (Fig. 2, arrows). Repeated coronary angiography did not show any significant stenosis of the coronary arteries (Fig. 3, arrows). Because chest pain and dyspnea persisted and cardiogenic shock was developed in spite of optimal medical treatment, intra-aortic balloon pump was applied and maintained for 3 months to maintain the adequate cardiac output and heart transplantation was performed finally. The explanted heart revealed amyloidosis in pathology. The gross finding showed that deposition of pale staining amorphous material in blood vessel and interstitium with degenerated myofibers (Fig. 4A) and microscopic findings stained with hematoxylin and eosin revealed myxoid and amorphous deposits in perivascular and interstitial spaces (Fig. 4B, arrowheads). Congo-red stain of the specimen revealed apple green birefringence under polarized microscopy (Fig. 5), a finding compatible with amyloid deposit. He has been well for 2 years after transplantation and his cardiac performance is New York Heart Association Functional Class I.

## Discussion

This case is of paramount importance for the following reasons. First, early clinical presentation of primary amyloidosis was typical angina symptoms, thus the patient was initially misdiagnosed as cardiac syndrome X. Second, a closer observation of the echocardiographic and ECG findings was sufficient to call into question the diagnosis of syndrome X. Moreover, it is interesting that our patient had a typical disease course with the later presentation of progressive heart failure after the initial presentation of exertional chest pain. Third, patient should inevitably undergone heart transplantation due to progressive heart failure despite of optimal medical treatment. Heart transplantation remains controversial because of the potential for amyloid deposition in the graft, or for multiple myeloma occurrence during follow up [5-7]. Low voltage waves in the limb and the precordial leads are the most common ECG finding in cardiac amyloidosis and are present in over 50% of patients with primary amyloidosis [8, 9]. According to the largest series of ECG findings in cardiac amyloidosis, the two main ECG abnormalities were the presence of low voltages and a pseudo-infarct pattern in 46% and 47% of patients, respectively [9]. Both ECG findings were present in 25% of patients [9]. Our patient had low voltages in the limb leads and poor R-wave progression in the precordial leads. Thickening of the left ventricular wall due to amyloid infiltration can be misdiagnosed as true left ventricular hypertrophy. The increased left ventricular wall thickness in cardiac amyloidosis, however, is associated with low voltages on the ECG and this feature is a specific finding for cardiac amyloidosis [9, 10]. Rahman et al. showed that a septal thickness > 1.98 cm combined with low voltages on ECG has a sensitivity of 72% and a specificity of 91% for the diagnosis of cardiac amyloidosis [9]. Doppler transmitral flow measurements showed a restrictive filling pattern with an E/A ratio > 2. Restrictive physiology in cardiac amyloidosis is attributed to amyloid infiltration, which results in a stiff myocardium [11]. Systolic function impairment is a late phenomenon [11]. Tissue Doppler imaging in cardiac amyloidosis has been shown to detect early diastolic dysfunction, even with minimal wall thickening [11]. Although congestive heart failure is the most common cardiac complication of amyloidosis, chest pain caused by an intramyocardial coronary artery obstruction with amyloid depositions is rarely observed [1-4]. One report stated that preexisting anginal symptoms before the onset of congestive heart failure occurred in 17% of patients with primary amyloidosis, although accumulation of amyloid in the intramyocardial coronary arteries was detected in 66% of these patients [12]. The previous published case reports describe the variable duration between the initial presentation of exertional angina and the later presentation of congestive heart failure as 21 months, 3 and 7 years, respectively [1-3]. In the present case, progressive congestive heart failure developed three years after initial presentation of syndrome X. Heart transplantation remains controversial [5-7]. There are a few patients who have received a cardiac transplant and shown variable survival [5-7]. Early postoperative results do not differ from other heart transplantation patients, but survival rates drop 30 months after transplantation in amyloidosis patients [7]. The survival of 7 patients with primary amyloidosis treated with heart transplantation and chemotherapy in the United Kingdom was 71% at 1 and 2 years, but 36% at 5 years [5]. Cardiac amyloidosis is a severe disease with a poor prognosis [1-4]. Therefore, cardiac amyloidosis should be considered when the patient has severe angina without any significant stenoses of the epicardial coronary arteries on coronary angiography and thickened left ventricular walls, with a restrictive filling pattern of the left ventricle combined with low voltages on the ECG.
